# Wireless ultrasonic power transfer using a pre-charged CMUT structure with a built-in charge storage capacitor

**DOI:** 10.1038/s41378-025-00902-w

**Published:** 2025-04-23

**Authors:** Muhammetgeldi Annayev, Feysel Yalçın Yamaner, Ömer Oralkan

**Affiliations:** Electrical and Computer Engineering, NC State University, 890 Oval, Drive, EB II, Raleigh, 27606 NC USA

**Keywords:** Electrical and electronic engineering, Electronic devices

## Abstract

Capacitive micromachined ultrasonic transducer (CMUT) technology is a potential candidate to implement an ultrasonic power receiver for implantable medical devices (IMDs) because CMUT technology employs photolithography-based microfabrication techniques amenable to miniaturization, integration with electronics, and biocompatibility. Pre-charged CMUTs operating in constant-charge mode eliminate the DC bias and this mode of operation is more suitable for ultrasound power transfer to IMDs. We designed and fabricated a novel pre-charged CMUT structure with a built-in charge storage capacitor. This new configuration features a floating electrode between the upper and lower electrodes. Charges are stored on this floating electrode prior to implantation by directly bringing the floating electrode into contact with the bottom electrode while applying a DC bias between the top and bottom electrodes of the CMUT. After pre-charging the CMUT, the charges are retained without any leakage, as confirmed by occasional measurements over the course of about two years. We have also demonstrated that this device allows operation without a DC bias and can be used as a power receiver in an IMD. In the presented design, the CMUT can be pre-charged at a desired precise charge level. The amount of trapped charge can be controlled by holding the floating electrode in contact with the bottom electrode by applying external ultrasound pressure and simultaneously maintaining a DC bias. The maximum received power was 10.1 mW, corresponding to a received power density of 3.1 mW/mm^2^, with a 14.5% efficiency. We have achieved an acoustic-to-electrical power conversion efficiency as high as 29.7% at lower input power levels.

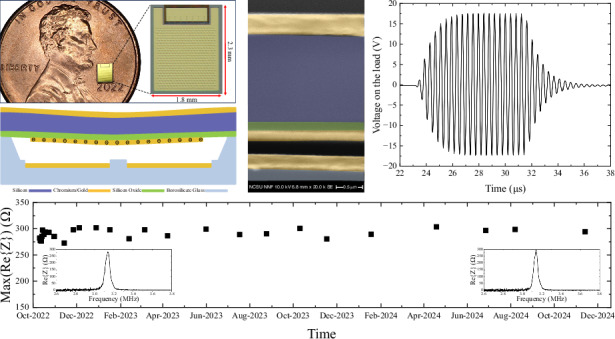

## Introduction

Implantable medical devices (IMDs) that support, monitor, or restore organ function have gained prominence in recent times. Transferring power to IMDs from an external source can extend the limited lifetime of implantable power sources^1^. An IMD can also be entirely powered externally by wireless power transfer if continuous operation is not required. There are different techniques for transferring power wirelessly to an implantable device, such as inductive coupling, capacitive coupling, electromagnetic coupling, and ultrasonic transmission and reception^2–4^.

Inductive coupling is one of the oldest wireless power transfer methods. In this approach, power is transferred by coupling two inductive coils through a magnetic field^5–7^. Powering the implanted devices with this method is challenging because of efficiency drops caused by the weak coupling resulting from misalignment or large separation between the coils^8–10^.

Capacitive coupling works based on the electric field coupling between two pairs of conducting plates^11,12^. The efficiency of this method is very sensitive to the separation between the external source and the implanted device. Capacitive coupling can be useful mainly in near-field power transfer applications. Safety of this approach should also be more carefully analyzed for transferring power through tissue.

Electromagnetic coupling is based on the principle of electromagnetic radiation^13,14^. Implanted devices have an antenna, and an external source transmits power with the radiated electric and magnetic fields. The electromagnetic link operates at GHz frequencies to make this approach suitable for small implants. As a result, this method suffers from higher transmission losses due to the higher absorption in tissue at these frequencies.

Uncontrolled electromagnetic energy that is absorbed in tissue has the potential to induce damage. In extreme cases, it can lead to fatal outcomes, particularly with the generation of substantial induced currents in critical tissue pathways like the heart or specific nerves^15^. All the mentioned methods have limitations related to their size, delivery range, or power density.

Ultrasound can deliver power to mm-scale devices in deep tissue. Ultrasound is safe, powerful, efficient, and compact. Thus, ultrasonic power transfer has attracted great attention for IMDs.

Energy harvesting from ultrasound requires converting mechanical vibrations into electrical energy. This can be achieved using triboelectric nanogenerators (TENGs), piezoelectric ultrasound transducers, or capacitive micromachined ultrasonic transducers (CMUTs).

TENGs work by contacting and separating two materials that exchange electrons to create surface charges. TENGs are cost-effective, lightweight, and flexible. However, they face challenges such as limited output power density and reduced durability^16,17^.

Piezoelectric ultrasound transducers use piezoelectric crystals to convert mechanical vibrations to electrical energy. They are recognized for their high sensitivity and efficiency. Nonetheless, their bulk ceramic processing makes miniaturization and integration with modern semiconductor electronics challenging. Moreover, many efficient piezoelectric ceramics contain lead, posing additional concerns for their use in IMDs^18^.

Capacitive micromachined ultrasonic transducers (CMUTs) have emerged as a viable alternative to TENGs and piezoelectric transducers. CMUTs offer benefits such as simplified batch fabrication, wide bandwidth, and potential for miniaturization and close integration with supporting electronic circuits, which make them attractive for use in implantable bioelectronics.

Efficient operation of CMUTs typically requires a DC bias voltage. For a CMUT that acts as a receiver operating under constant DC bias, the output current depends on the amplitude and frequency of the incoming ultrasound wave and the amplitude of the DC bias voltage. In the absence of a DC bias voltage or embedded charges, the CMUT will not produce any electrical current. Providing a DC bias voltage to the CMUT typically requires an additional battery. The requirement for a DC bias voltage can be eliminated by employing a CMUT with a fixed embedded charge to allow operation in constant-charge mode, in which the incoming pressure wave puts the thin plate in motion, which in turn modulates the capacitance. With a fixed amount of charge in the capacitor, the capacitance changes will result in voltage changes across the capacitor. As no external bias voltage is needed, this will be a more suitable mode of operation for transferring power to implantable devices.

Previously, we reported a pre-charged CMUT incorporating a metallic floating electrode, that was sandwiched between silicon oxide and silicon nitride insulation layers^19^. Charge transfer was achieved by Fowler-Nordheim (FN) tunneling through the silicon oxide layer into the metal floating gate. This device was used by connecting it to a custom integrated circuit to demonstrate wireless power transfer by using ultrasound^20^.

There are also other previously reported pre-charged CMUT designs^21–23^. In a paper by Park et al.^21^, the charges were trapped at the interface between silicon dioxide and silicon nitride layers. In a paper by Ho et al.^22^, the charges were trapped in a floating silicon island. In a paper by Saccher et al.^23^, the charges were trapped in a floating Al_2_O_3_ layer. In all the previous work, FN tunneling was used to transfer charges through an oxide layer. FN tunneling requires a high electric field, and it is a quantum phenomenon that is hard to control^24^. To address this challenge, we have developed a CMUT structure to bring the floating electrode in direct contact with one of the fixed electrodes and used external pressure to control the contact duration so that the amount of charge transferred to the floating electrode can be controlled as well.

## Results

**Fig. 1:**
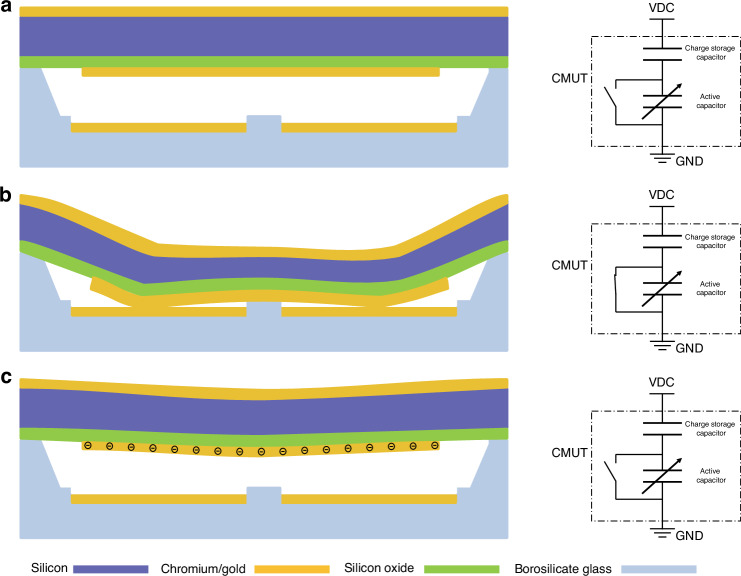
Charge trapping process onto the floating electrode. **a** Initial stage with no charge on the floating electrode. **b** Floating electrode contacting the bottom electrode due to the pull-in (collapse) or external mechanical force. **c** Floating electrode with negative charge (Q) after charge writing

We have designed and fabricated a novel pre-charged CMUT structure featuring a built-in charge storage capacitor. The design includes a floating electrode positioned on the surface of a dielectric layer beneath the vibrating plate (Fig. 1a). For trapping charges on the floating electrode, we establish a physical contact between the floating electrode and the bottom electrode while simultaneously applying a DC bias voltage between the top and bottom electrodes of the CMUT (Fig. 1b). There are different ways to establish the contact as we explained in detail in the Discussion Section.

One way to bring the floating electrode in contact with the bottom electrode is to apply a DC bias voltage that is greater than the pull-in (collapse) voltage of the transducer causing the thin plate to become unstable and spontaneously collapse on the bottom electrode. During the contact, the active capacitor will be shorted, and charges will start accumulating on the charge storage capacitor. The charging process will continue until the contact is broken or the storage capacitor is fully charged up to the applied DC voltage. When the floating electrode contacts the bottom electrode, charges accumulate on the floating electrode, causing the electric field in the active capacitor to decrease. This reduction leads to a decrease in the electrostatic force acting on the plate. If the collapsed plate is solely held down by the electrostatic force resulting from the static DC bias, the floating electrode will remain in contact as long as the electrostatic force is greater than the mechanical restoring force. Once the mechanical force exceeds the electrostatic force, the plate will snap back (Fig. 1c). The amount of trapped charge depends on the contact duration and current flow during contact. So, the electrical and mechanical time constants are the main determining factors for the number of trapped charges.

Once charges are captured, we anticipate no leakage from the floating electrode to the bottom electrode, due to the vacuum gap. Additionally, a glass spacer is created in the center of the cells to act as a safeguard, preventing inadvertent short-circuits between the floating electrode and the bottom electrode during operation. In a prior study, we showed the capability of designing and fabricating spacers with different shapes inside CMUT cells, demonstrating the potential to operate CMUTs in collapse mode with improved reliability, i.e., reduced unintended charging and dielectric breakdown risk^25^.

### Measurements in air

First, we measured the electrical input impedance of the fabricated transducers in air at different DC bias levels, which were lower than the collapse voltage. Devices exhibited a resonance at 3.2 MHz observed as a peak in the real part of the electrical input impedance when an external DC bias was applied. No resonance was observed at zero bias. This means no charges were trapped in the floating electrode if the plate did not collapse. At the same time this was a confirmation that no charges were trapped during the fabrication process.

**Fig. 2:**
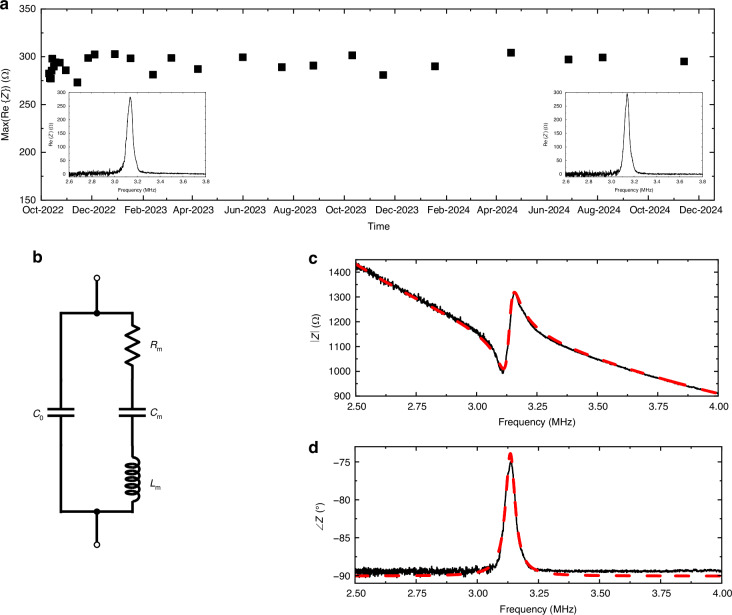
**a** Measurements of the impedance peak after charging, without applying a DC bias voltage – Inset shows the real part of the electrical input impedance measured at different times; **b** Butterworth–Van Dyke equivalent circuit model (C_0_ = 43.9 pF, R_m_ = 4.1 kΩ, C_m_ = 0.19 pF, L_m_ = 13.7 mH); **c** Magnitude plots of measured (solid black line) and fitted (dashed red line) electrical input impedance of pre-charged CMUT; **d** Phase plots of measured (solid black line) and fitted (dashed red line) electrical input impedance of pre-charged CMUT

After checking the impedance at lower DC bias voltages, we increased the DC bias to collapse the plate and trap charges onto the floating electrode. We applied a DC bias up to 40 V. The collapse voltage was 35 V. So, we ensured the floating electrode contacted the bottom electrode. Then, we reduced the bias voltage to 0 V, and we observed a peak in the real part of the electrical input impedance (Fig. 2). This meant charges were trapped in the floating electrode. After pre-charging the CMUT, we measured the electrical input impedance without applying any DC bias voltage at different times for more than two years (Fig. 2a). The peak value of the real part of the input impedance was observed to be stable, indicating the trapped charges were retained without any leakage for over two years.

The measured electrical input impedance of the pre-charged CMUT is modeled using a Butterworth–Van Dyke equivalent circuit model (Fig. 2b). In this model, the inductance (L_m_), capacitance (C_m_), and resistance (R_m_) represent the mechanical components of the CMUT. Specifically, the inductance (L_m_) corresponds to the mass of the plate, the capacitance (C_m_) represents inverse of the stiffness of the plate, and the resistance (R_m_) accounts for the motional resistance arising from energy radiated into the medium and coupled to the substrate.

Magnitude and phase plots of the measured (solid black line) electrical input impedance of the pre-charged CMUT is fitted (dashed red line) using the Butterworth-Van Dyke equivalent circuit with component values, C_0_ = 43.9 pF, R_m_ = 4.1 kΩ, C_m_ = 0.19 pF, and L_m_ = 13.7 mH (Fig. 2c, d).

### Measurements in immersion

**Fig. 3:**
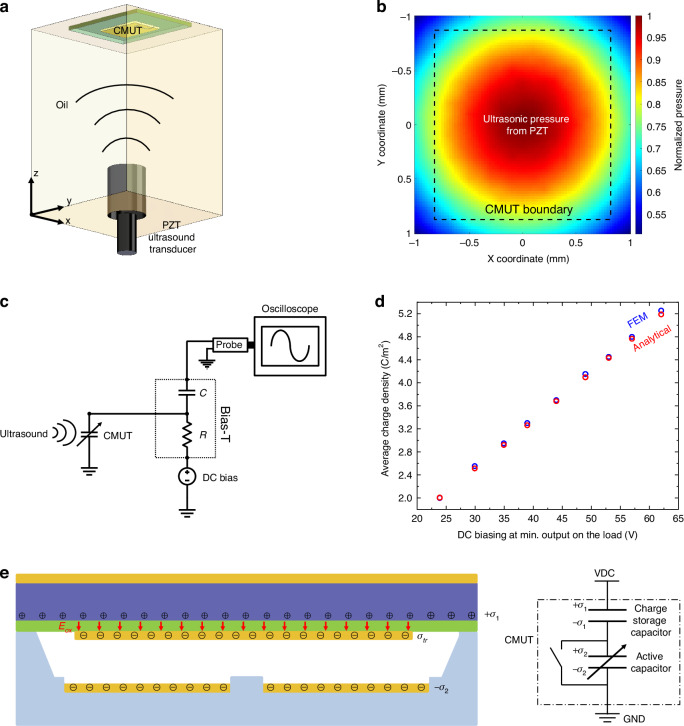
**a** Illustration of the experimental setup including the CMUT receiver aligned with the focused piezoelectric transducer set as a transmitter; **b** Pressure pattern generated by the focused piezoelectric transducer on the surface of the CMUT; **c** Schematic of the setup for charge trapping using external ultrasound pressure and DC biasing; **d** DC biasing at minimum output on the passive probe load vs. trapped average charge density on the floating electrode; **e** Illustration of charges and electric field for the analytical calculation and two capacitor model of the pre-charged CMUT

We wire-bonded a CMUT element onto a printed circuit board (PCB) for further characterization in immersion. We chose to conduct the measurements in oil because the bond wires and the transducer surface were not electrically insulated. We set up an external focused piezoelectric ultrasound transducer (V325, Olympus NDT, Waltham, MA) in oil as a transmitter by aligning it with the CMUT (Fig. 3a). The transducer has a focal distance of 19 mm; however, characterization was performed at 35 mm distance to increase the average pressure on the surface of the CMUT. The pressure field generated by the focused piezoelectric transducer on the surface of the CMUT was characterized using a calibrated hydrophone (Model HGL-0200, Onda Corporation, Sunnyvale, CA, USA) (Fig. 3b). To increase the number of charges on the floating electrode, an external ultrasonic pressure was applied by the focused transducer to initiate physical contact between the floating electrode and the bottom electrode. The center frequency of the CMUT in immersion is 2.45 MHz, which is lower than its resonant frequency in air, as expected. We applied the external ultrasound pressure at a frequency of 2.45 MHz, matching it with the center frequency of the CMUT in immersion. This was done while biasing the CMUT with an external DC voltage (Fig. 3c). We used a digital oscilloscope (MSO9064A, Agilent Technologies, Santa Clara, CA) with a 10:1 passive probe (N2863B, Keysight, Colorado Springs, CO) and a bias-T circuit to measure the voltage at the output of the CMUT during the charging process.

The trapped charges on the floating electrode oppose the field resulting from the externally applied bias voltage and therefore decrease the net electric field in the cavity. In this part of our experiments, we decreased the bias voltage until the output voltage of the CMUT observed through the passive probe was minimal. At this point, we assumed that the electric field is zero in the vacuum gap, attributed to the presence of trapped charges and the cancellation of the electric field in the vacuum gap by the external DC bias voltage with opposing polarity. Hence, despite the presence of mechanical vibration, the output power remains close to zero.

We performed FEM simulations (ANSYS v19.2, ANSYS Inc., Canonsburg, PA) with coupled field analysis as well as analytical calculations to estimate the average trapped charge density on the floating electrode by using DC voltages applied to make the net field in the cavity zero in the described experiment (Fig. 3d).

For coupled field analysis (FEM simulations), we applied the DC bias voltages to the top and the bottom electrodes in the simulations. Then we placed different numbers of charges on the floating electrode to get zero/minimal net electric field in the cavity as well as minimal deflection of the plate.

For analytical calculations, we represented the pre-charged CMUT using a circuit model with two series parallel-plate capacitors with trapped charge at the node at which they are connected to each other (Fig. 3e).

The trapped charge density on the floating electrode in relation to the charge density of the top and bottom electrodes will then be:

1 $${\sigma }_{{tr}}={\sigma }_{2}-{\sigma }_{1}$$

where ${\sigma }_{{tr}}$ is trapped average charge density on the floating electrode; and ${\sigma }_{1}$ and ${\sigma }_{2}$ are surface charge densities on top and bottom electrodes, respectively. Since the net electric field in the cavity is zero/minimal:

2 $${\sigma }_{2}=0$$

3 $${\sigma }_{{tr}}=-{\sigma }_{1}$$

The entire DC voltage appears on ${C}_{1},$ the charge storage capacitor, Then,

4 $${V}_{{dc}}\cdot {C}_{1}={Q}_{1}$$

5 $${V}_{{dc}}\cdot \frac{{\varepsilon }_{0}\cdot {\varepsilon }_{{ox}}\cdot {Area}}{{t}_{{ox}}}={\sigma }_{1}\cdot {Area}$$

where ${\varepsilon }_{0}$ is the vacuum permittivity; ${t}_{{ox}}$ is thickness of the oxide insulating layer; and ${\varepsilon }_{{ox}}$ is the dielectric constant of the oxide layer. From Eq. 3 and Eq. 5, charge surface density of the top plate, ${\sigma }_{1}$, and the floating electrode, ${\sigma }_{{tr}}$, will become:

6 $${\sigma }_{1}=\frac{{V}_{{dc}}{\cdot \varepsilon }_{0}\cdot {\varepsilon }_{{ox}}}{{t}_{{ox}}}$$

7 $${\sigma }_{{tr}}=-\frac{{V}_{{dc}}{\cdot \varepsilon }_{0}\cdot {\varepsilon }_{{ox}}}{{t}_{{ox}}}$$

**Fig. 4:**
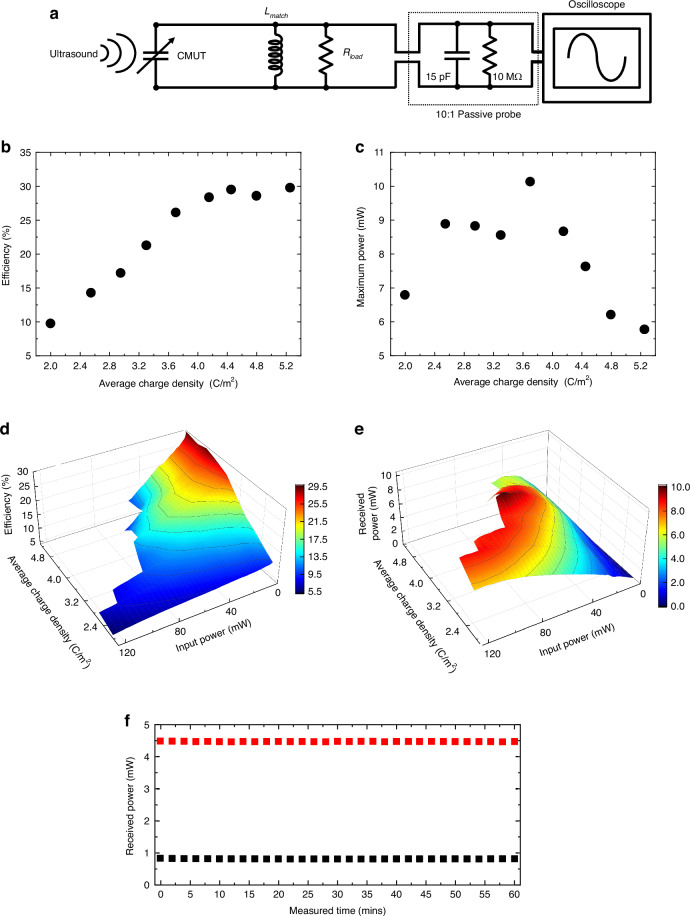
**a** Schematic of ultrasound power transfer measurement setup; **b** Measured acoustic-to-electrical power conversion efficiency as a function of different trapped charge densities for low input power ( < 1 mW); **c** Measured maximum power on the load as a function of different trapped charge densities; **d** Measured acoustic-to-electrical power conversion efficiency as a function of different trapped charge densities and input power levels; **e** Measured power on the load as a function of different trapped charge densities and input power levels; **f** Measured power on the load as a function of elapsed time, measured at two different power levels [black – low power (0.8 mW), red – high power (4.5 mW)]

After trapping charges on the floating electrode and calculating the average charge densities, we have demonstrated wireless ultrasound power transfer for each charge level and measured the received power by the CMUT (Fig. 4a). We connected a 68-μH matching inductor in parallel with the pre-charged CMUT to resonate out the total shunt capacitance of the pre-charged CMUT and the 15-pF capacitance of the passive probe at the 2.45 MHz operating frequency. The matching inductor value was set based on the measured imaginary value of the input impedance and the frequency of operation. We connected a 14.93 kΩ resistance (15 kΩ ± 5%) as a load for the pre-charged CMUT. The pre-charged CMUT demonstrated maximum power output when terminated with this specific resistance value among various resistances tested.

The total ultrasonic input power to the CMUT was calculated using the pressure pattern shown in Fig. 3b. The ultrasonic power was integrated over the entire transducer surface, and then divided by the total area to obtain the average intensity. The acoustic power integrated on the CMUT surface is the input power to the CMUT. The average electrical power delivered to the load is the output power of the receiver.

We measured the efficiency of power reception by the CMUT by calculating the ratio of the average output power on the load to the total ultrasonic power on the surface of the CMUT for each average trapped charge density level (Fig. 4b). Firstly, we observed that the efficiency increased by trapping more charges to the floating electrode. However, it began to saturate after reaching 29%. The highest efficiency measured was 29.7%.

We also measured the maximum output power on the load for each average trapped charge density level (Fig. 4c). The maximum output power increased with increasing average trapped charge density initially. Although trapping an increased number of charges enhances the power conversion efficiency it would not always increase the maximum output power on the receiver. A higher charge density produces a stronger electric field in the cavity and at the same time a greater plate deflection which means the floating electrode gets closer to the bottom electrode. As a result, the available gap for plate’s AC displacement decreases leading to reduced maximum output power.

Measured acoustic-to-electrical power conversion efficiency and received power as a function of different input power levels and trapped charge densities are given in Fig. 4d, e. These graphs allow for the selection of the required charge density and input power level for specific power needs of a particular IMD.

A maximum peak-to-peak pressure of 850 kPa was measured at the center of the pressure field that was used as an input to CMUT, corresponding to a maximum applied acoustical input power of 120 mW, generated by the focused piezoelectric transducer on the surface of the CMUT (Fig. 3b). The mechanical index (MI) was 0.27, and the average intensity was 60 mW/mm². The FDA limit for acoustic exposure to human tissue in diagnostic imaging applications is stated for the global maximum derated spatial-peak pulse-average intensity (I_SPPA_) as 1900 mW/mm², and the limit for the mechanical index is 1.9. All the parameters used in the presented experiments are well below the FDA safety limits.

To test any variability of the received power, we performed measurements for long periods of time in burst mode. Ultrasound power was transmitted every 10 ms in a 20-cycle, 2.45-MHz tone burst for 60 mins at two different power levels [black – low power (0.8 mW), red – high power (4.5 mW)] (Fig. 4f). No variations were observed in the received power level during the 60 min test.

**Fig. 5:**
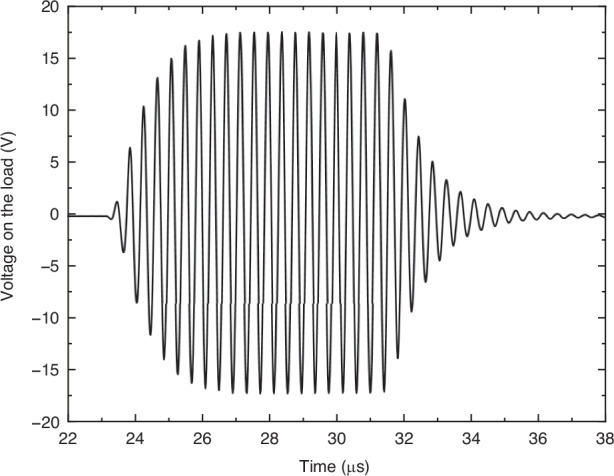
The maximum measured voltage signal on the load corresponding to a received electrical average power of 10.1 mW

The maximum voltage signal measured on the load was 34.7 V_pp_ (Fig. 5), which corresponds to 10.1 mW average electrical power received with 14.5% efficiency. This was the highest received average power on the load. An electrical power budget of 1 mW is generally sufficient for most IMD applications, including pacemakers, cardiac defibrillators, neurostimulators, and drug pumps^26^. The measured average electrical power of 10.1 mW, which is substantially higher than 1 mW, suggests that the pre-charged CMUT is well-suited for these IMDs.

A comparison of results accomplished in this work and results reported by others earlier is shown in Table 1.

**Table 1:** Comparison of results accomplished in this work and others’ earlier results

	^16^	^32^	^33^	^34^	^35^	^36^	^37^	^38^	This work
Frequency (MHz)	0.02	1	0.673	1	1.85	3	2	5.85	2.45
Transducer Type	TENG	PZT	PZT	PZT	PZT	PMUT	PMUT	CMUT	CMUT
Max. Efficiency (%)	—	20	27	1.6	3.4	0.236	0.7	47	29.7
Max. Output Power (mW)	0.009	2100	100	28	0.065	0.019	0.07	0.78	10.1
Active Device Surface Area (mm^2^)	400	706	177	50	2.81	2.55	11	6.3	3.24
Received Power Density (mW/mm^2^)	0.0002	2.97	0.57	0.56	0.023	0.008	0.006	0.12	3.12

## Discussion

As we have mentioned previously, for charge writing into the CMUT we need to establish contact between the floating electrode and the bottom electrode. This contact can be initiated in different ways. The first of these is with electrostatic force by applying a DC bias voltage higher than the pull-in (collapse) voltage of the device. In a voltage controlled electrostatic actuator, a stability of the equilibrium exists between the electrostatic force pulling the plate down and the spring force pulling it up. The equilibrium gap decreases with increasing voltage. At a specific voltage, the stability of the equilibrium is lost and the plate collapses on the bottom electrode^27^. In the structure we presented, this pull-in brings the floating electrode and the bottom electrode in contact, which initiates the transfer of charges onto the floating electrode. As this charge transfer continues, the electrostatic force holding the plate down decreases and at some point, the plate releases and charge transfer stops. The eventual number of charges trapped on the floating electrode will depend on how fast the charges move and how quickly the plate releases. In other words, the electrical and mechanical time constants will determine the final charge density on the floating electrode. If one can prolong the duration of the contact, the floating electrode could be possibly charged up to the applied external DC bias voltage. One way of prolonging the contact and preventing the plate from releasing is to apply external mechanical forces on the plate. This can be accomplished by placing the vacuum sealed CMUT in a highly pressurized chamber. Alternatively, using an external ultrasound wave reflected off the CMUT surface would exert a static radiation force on the plate, or resonate the plate with a high displacement amplitude to facilitate periodic contact between the floating and fixed bottom electrodes.

This novel pre-charged CMUT design holds potential for further enhancement in both efficiency and power. Parameters, such as gap height, cell dimensions, insulating layer and silicon plate thicknesses, and fill factor can be further optimized. Increasing the vacuum gap height provides flexibility for writing a greater number of charges and achieving higher received power. The choice of insulating layer thickness should align with the targeted charge density to mitigate potential charge leakage. Additionally, we have flexibility to design the operating frequency by modifying cell dimensions and the thickness of the silicon plate based on the application’s requirements. Incorporating a higher fill factor in the CMUT design contributes to further improved efficiency.

The single element pre-charged CMUT used in the presented study requires fine directional alignment. We can design and fabricate 2D pre-charged CMUT arrays to eliminate the requirement for fine directional alignment of the implanted device to an external transducer. Each element of that 2D pre-charged CMUT array can have an individual power collector of harvested energy by bonding them with a custom designed integrated circuit (IC) and all the power can be added together to achieve a higher level of received power^28^.

## Methods

### Design and fabrication of devices

Finite element modeling (FEM) (ANSYS v.19.2, ANSYS Inc., Canonsburg, PA, USA) was used to design the presented pre-charged CMUT structure. There are previously reported papers^25,29^, which explain in detail how to use FEM simulations to design CMUTs for achieving desired metrics such as collapse voltage, bandwidth, output pressure, and receive sensitivity. We used static, harmonic, and transient analyses to optimize the collapse voltage and the frequency of operation. Coupled-field analysis was used to find the optimal floating electrode dimensions.

CMUT design was finalized by defining device dimensions, such as plate thickness, gap height and diameters of the cell, bottom electrode, and floating electrode.

**Fig. 6:**
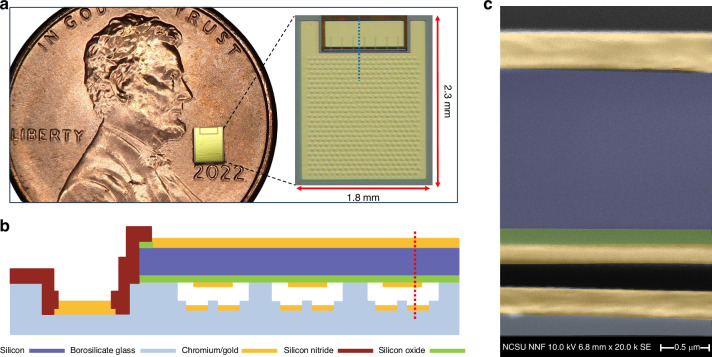
**a** Photograph of the fabricated pre-charged CMUT, **b** Cross-sectional schematic view of three cells, [through the blue dashed line in panel **a**], **c** Cross-sectional view in SEM [through the red dashed line in panel **b** – pseudocolor added for marking different layers]

Devices were fabricated by using an aligned anodic wafer bonding method on a 100-mm borosilicate glass wafer (Borofloat33, Schott AG, Jena, Germany). The fabrication process was briefly explained in a paper by Annayev et al.^30^, which is based on a fabrication process that we previously developed by using only three masks and anodic bonding^31^ The fabricated device has a total of five hundred cells connected electrically in parallel to form a transducer element, 2.3 mm by 1.8 mm in size (Fig. 6a). Cross-sectional view of fabricated devices is shown in Fig. 6b.

Device dimensions are given in Table 2. The glass spacer placed in the middle of the cell is for preventing accidental contact of the floating electrode with the bottom electrode during the power reception. This is critical to retain the charge in the floating electrode as such an accidental contact would cause an electrical short between the floating electrode and bottom electrode resulting in discharging the storage capacitor.

**Table 2:** Device dimensions

Cell diameter	70 µm
Number of cells	500
Bottom electrode diameter	54 µm
Floating electrode diameter	48 µm
Spacer diameter	6 µm
Silicon plate thickness	1.5 µm
Insulating oxide layer thickness	0.2 µm
Spacer height	0.03 µm
Gap height	0.19 µm

Floating electrodes are in vacuum cavities, and they are isolated from the external environment to prevent any possible charge leakage after charge trapping. The total gap height, from the surface of the floating electrode to the surface of the bottom electrode, is 190 nm as seen in the cross-sectional SEM image (Fig. 6c).

## Conclusion

We successfully designed and fabricated a novel pre-charged CMUT structure with a built-in charge storage capacitor on a borosilicate glass substrate. By trapping different amounts of charge in the built-in storage capacitor, we demonstrated wireless power transfer at different acoustical input power levels. The device achieved a maximum received power of 10.1 mW, corresponding to a power density of 3.1 mW/mm², with an efficiency of 14.5%. Additionally, we demonstrated an acoustic-to-electrical power conversion efficiency as high as 29.7% at lower input power levels.

We showed that the trapped charges remained stable for over two years, as confirmed through periodic electrical input impedance measurements. We also tested the reliability and variability of the received power by performing ultrasound wireless power transfer in burst mode with 20 cycles every 10 ms for 60 min. The results showed less than 1% fluctuation.

The maximum applied peak-to-peak pressure on the surface of the CMUT was 850 kPa. The corresponding mechanical index (MI) was 0.27, and the average intensity was 60 mW/mm². The FDA limits for the global maximum derated spatial-peak pulse-average intensity (I_SPPA_) and the mechanical index are 1900 mW/mm² and 1.9, respectively. All parameters used in the presented experiments were well below the FDA safety limits.

In the future, we plan to achieve a fully integrated compact system by combining the pre-charged CMUT with a custom IC. The system will be tested through in vitro and in vivo experiments.
